# Increasing Contact with Hepatitis E Virus in Red Deer, Spain

**DOI:** 10.3201/eid1612.100557

**Published:** 2010-12

**Authors:** Mariana Boadella, Maribel Casas, Marga Martín, Joaquín Vicente, Joaquim Segalés, José de la Fuente, Christian Gortázar

**Affiliations:** Author affiliations: Instituto de Investigación en Recursos Cinegéticos (CSIC-UCLM-JCCM), Ciudad Real, Spain (M. Boadella, J. Vicente, J. de la Fuente, C. Gortázar);; Centre de Recerca en Sanitat Animal, Barcelona, Spain (M. Casas, M. Martín, J. Segalés);; Universitat Autònoma de Barcelona, Barcelona (M. Martín, J. Segalés);; Oklahoma State University, Stillwater, Oklahoma, USA (J. de la Fuente)

**Keywords:** Viruses, epidemiology, hepatitis E, red deer, reservoir, wildlife, zoonosis, Spain, dispatch

## Abstract

To describe the epidemiology of hepatitis E virus (HEV) in red deer in mainland Spain, we tested red deer for HEV RNA and antibodies. Overall, 10.4% and 13.6% of serum samples were positive by ELISA and reverse transcription–PCR, respectively. The increasing prevalence suggests a potential risk for humans.

Hepatitis E virus (HEV) is the only member of the *Hepeviridae* family (*1*). Four major genotypes of HEV have been recognized: genotypes 1 and 2 are restricted to humans and associated with epidemics in developing countries; genotypes 3 and 4 are zoonotic in developing and industrialized countries. Wild and domestic animals are being identified as potential HEV reservoirs (*1**–**3*).

Studies on wild sika deer (*Cervus nippon*) have detected low prevalence rates for HEV, which suggests that sika deer are accidental hosts for the virus (*4**,**5*), despite the transmission link discovered between them and HEV in Japan (*3*) that raised awareness of the possibility that game animals transmit HEV (*2*). In Europe, information about HEV infection in wild ruminants is limited to reports suggesting that roe deer (*Capreolus capreolus*) and red deer (*Cervus elaphus*) can act as HEV hosts (*6**–**8*). Except for these limited studies, no large-scale surveys have been conducted of HEV epidemiology in wild cervids. In Spain, the relatively high HEV seroprevalence detected in domestic pigs and wild boar suggests that HEV infection is probably widespread (*9*).

Red deer density, distribution, and hunting harvest are increasing throughout Europe (*10*). In Spain, the high densities recorded (*11*) indicate that red deer are an important source of game meat. This scenario emphasizes the need for a better understanding of the epidemiology of HEV in game populations in Spain.

Our goals were to describe the epidemiology and time trends of HEV in red deer in peninsular (mainland) Spain by serologic testing and PCR. On the basis of previous results on wild boar (*9*), we hypothesized that red deer would show widespread contact with HEV in Spain.

## The Study

Serum samples from 968 Iberian red deer were collected during 2000–2009. These samples came from hunter-harvested red deer in 21 wild or semifree ranging populations (892 deer) and from 2 farms (76 deer). Sampling sites were representative of a variety of habitats and climates, which can be simplified into 5 different bioregions (Figure) (*12*). Sampling sites were grouped into 7 areas and 2 red deer farms (Table; Figure). Sex and age of deer were recorded. Management conditions of red deer were classified as open (no fencing and no management, 9 sites), fenced (fencing and artificial feeding, 12 sites), and farmed (livestock-like management, 2 farms). To analyze time trends, we classified samples collected during 2000–2005 as time 1 and those collected during 2006–2009 as time 2. Only sites where sampling occurred in both periods and with comparable sampling sizes were included in the time-trend analysis.

**Figure:**
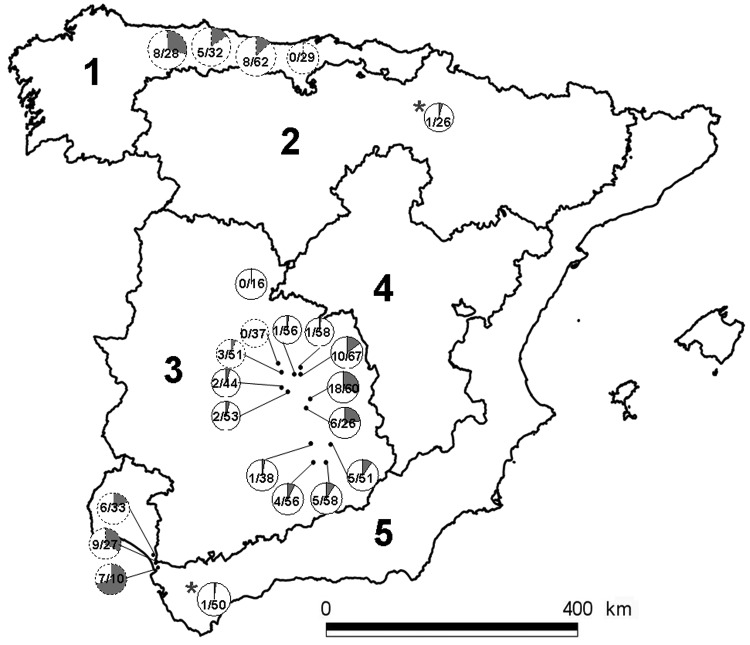
The 5 peninsular bioregions (nos. 1–5) and the 21 sampling sites, Spain. Pie charts indicate local prevalence (in gray). Numbers indicate positive animals/sampled animals. Broken line borders indicate open sites; solid lines indicate fenced estates; asterisks indicate the 2 red deer farms.

**Table:** IgG serologic results for HEV and RT-PCR results in different regions and 2 red deer farms, Spain*

Region	No. sites	No. samples	No. seropositive	Prevalence, % (95% CI)	RT-PCR
Cantábrico Occidental	3	122	21	17.2 (11.4–24.9)	2/14
Cantábrico Oriental	1	29	0	0.0 (0.0–11.5)	0
Sistema Central	1	16	0	0.0 (0.0–20.8)	0
Montes de Toledo	7	366	19	5.2 (3.2–8.0)	2/18
Valle del Guadiana	2	86	22	25.6 (17.3–35.9)	5/13
Sierra Morena	4	203	15	7.4 (4.3–11.9)	1/14
Doñana	3	70	22	31.4 (21.3–43.5)	1/21
Cádiz†	1	50	1	2.0 (0.1–10.6)	0
Navarra†	1	26	1	3.8 (0.2–18.8)	0
Total	23	968	101	10.4 (8.62–12.53)	11/81

*Ig, immunoglobulin; HEV, hepatitis E virus; CI, confidence interval; RT-PCR, reverse transcription–PCR. †Red deer farms.

Serum samples were tested for HEV immunoglobulin (Ig) G by using ELISA as described (*4**,**13*), except for including protein G horseradish peroxidase (Sigma Chemical, St. Louis, MO, USA) as a conjugate, as in previous studies of red deer (*12*). Anti-HEV–positive serum was obtained from domestic swine that were positive for HEV by ELISA and reverse transcription–PCR (RT-PCR). Anti-HEV–negative serum was obtained from previous studies (*14*) and negative controls from HEV-negative cattle (*13*). Results were expressed as the percentage of optical density (% OD) by using the formula [% OD = 100 × sample OD/sum of negative controls OD]. Serum samples with % OD values >100% were considered positive.

For the RT-PCR, 81 serum samples were randomly selected and analyzed. Viral RNA was extracted from 150 mL of serum with Nucleospin RNA virus kit (Macherey-Nagel, Düren, Germany) by following the manufacturer’s instructions. HEV was detected by using a seminested RT-PCR as described (*14*). In each run, negative and positive controls were added.

Eight HEV RT-PCR–positive samples were sequenced. HEV sequences were identified by using the BLAST algorithm (www.ncbi.org) against HEV sequences available in GenBank (on January 25, 2010). Sequences were deposited in the GenBank database under accession nos. HM113373 and HM113374.

Sterne exact method was used to estimate apparent prevalence confidence intervals (CIs). χ^2^ tests were used to analyze the association of age, sex, sampling site, and management conditions with serologic and RT-PCR results. Association between seropositivity and HEV RNA in the serum was also analyzed by using Pearson χ^2^ test. Differences were considered statistically significant at p<0.05.

Overall, 101 (10.4%, 95% CI 8.6–12.5) serum samples were positive for IgG (Table). HEV seroprevalence did not differ significantly between sex (χ^2^ = 0.894, 1 df, p>0.05) and age classes (χ^2^ = 12.436, 3 df, p>0.05). Seroprevalence in time 2 (12.2%, 95% CI 9.8–15.0) was significantly higher than seroprevalence in time 1 (7.5%, 95% CI 5.1–10.8) (χ^2^ = 5.181, 1 df, p<0.05). Local IgG seroprevalences ranged from 0 (95% CI 0–20.8) to 31.4% (95% CI 21.3–43.5; Figure). IgG seroprevalence differed significantly by management types (χ^2^ 6.876, 2 df, p<0.05), with higher values in open (14.9%, 95% CI 11.3–19.4) than in fenced (9.1%, 95% CI 7.0–11.7) and farmed (2.6%, 95% CI 0.5–9.0) areas.

Eleven (13.6%, 95% CI 7.4–22.7) of 81 samples were RT-PCR positive. Local viral RNA prevalence ranged from 4.5% (95% CI 2.4–22.21 to 38.5% (95% CI 16.6–65.8; Table). HEV prevalence did not differ significantly by geographic area and management type.

Sequence analysis indicated that all deer sequences from this study belonged to genotype 3. Seven samples belonging to sequence HM113374 shared 99% nucleotide identity with domestic swine strains from Spain. One sample, sequence HM113373, showed similarity (91%) with a strain from acute hepatitis E in a person in Marseille, France, according to GenBank.

## Conclusions

Our findings of HEV infection confirm that HEV circulates actively among red deer in the Iberian Peninsula, as described for wild boar (*9*). Red deer can be infected with HEV (*7**,**8*), and the results of our large serosurvey in this species in Europe show an increasing prevalence trend in the last decade.

de Deus et al. found higher IgG seroprevalences in estates with higher wild boar densities (*9*). However, in the present study, mean seroprevalence rates were lowest in red deer farms, where densities were the highest and red deer had no contact with wild boar or domestic swine. In contrast, the highest seroprevalence rates were reported in open areas where contact with suids may have occurred. However, wild boar densities also are high in fenced hunting estates (*15*), and HEV antibody prevalence rate was intermediate in deer from these sites. These differences could indicate that red deer may need a source of infection and thus act as spillover hosts more frequently than as true reservoirs.

Presence of HEV RNA in 13% of deer serum implies that deer represent a risk for zoonotic transmission, and consequently, handling of live animals and carcasses is a risk activity. Red deer are infected with HEV at lower rates than are wild boar and domestic pigs but may act as a potential source of HEV infection in humans. Further studies are needed to fully elucidate the epidemiology of HEV in wildlife and the foodborne zoonotic transmission risks.
